# Cognitive behavioural therapy for social anxiety disorder in people with bipolar disorder: a case series

**DOI:** 10.1186/s40345-023-00321-8

**Published:** 2024-01-05

**Authors:** Barbara Pavlova, Emma Warnock-Parkes, Martin Alda, Rudolf Uher, David M. Clark

**Affiliations:** 1 Department of Psychiatry, Dalhousie University, 5909 Veterans’ Memorial Lane, Halifax, NS B3H 2E2 Canada; 2 Nova Scotia Health, Halifax, NS Canada; 3 Department of Experimental Psychology, University of Oxford, Oxford, UK; 4 King’s College London, London, UK

**Keywords:** Bipolar disorder, Social anxiety disorder, Cognitive therapy

## Abstract

**Background:**

Social anxiety disorder increases the likelihood of unfavourable outcomes in people with bipolar disorder. Cognitive behavioural therapy (CBT) is the first-line treatment for social anxiety disorder. However, people with bipolar disorder have been excluded from the studies that this recommendation is based on.

**Method:**

We completed a case series to obtain initial data on whether CBT is an acceptable, safe, and effective treatment for social anxiety disorder in people with bipolar disorder.

**Results:**

Eleven euthymic participants with bipolar disorder attended up to sixteen treatment and three follow-up sessions of CBT for social anxiety disorder. Participants attended on average 95% of the offered CBT sessions. No adverse events were reported. Participants’ mean score on the Social Phobia Inventory decreased from 46.5 (SD 6.6) before the treatment to 19.8 (SD 11.9) at the end of the sixteen-session intervention and further to 15.8 (SD 10.3) by the end of the 3-month follow-up. This degree of improvement is equivalent to the effect observed in studies of CBT for social anxiety disorder in people without severe mental illness.

**Conclusions:**

This case series provides preliminary evidence that CBT is acceptable, safe, and effective for treating social anxiety disorder in people with bipolar disorder during euthymia. A randomized controlled trial is needed to confirm these findings, and to establish whether treatment for social anxiety disorder improves the course of bipolar disorder.

## Introduction

Bipolar disorder affects 1–2% of the population (Merikangas et al. 2011; Clemente et al. 2015; Moreira et al. 2017; Humpston et al. 2021). Effective treatments are available (Yatham et al. 2018) but treatment outcomes are mixed. While some people with bipolar disorder return to their premorbid level of functioning, many experiencelongstanding functional impairment (MacQueen et al. 2001; Judd et al. 2008; Marwaha et al. 2013). A diagnosis of bipolar disorder shortens one’s life by almost 13 years (Chan et al. 2022).

One predictor of unfavourable course of illness in people with bipolar disorder is a comorbidity with anxiety disorders, which is associated with negative outcomes, including an overall higher severity of bipolar disorder (Pavlova et al. 2018), shorter time to relapse (Lorenzo-Luaces et al. 2018) and suicide attempts (Simon et al. 2007; Amuk and Patel 2020). This comorbidity is common. Almost a half of people with bipolar disorder meet diagnostic criteria for an anxiety disorder in their lifetime (Pavlova et al. 2015). This is not simply anxiety occurring during depressive episodes, because a third of individuals with bipolar disorder, still meet diagnostic criteria for an anxiety disorder during euthymia (Pavlova et al. 2017).

Social anxiety disorder is one of the most common anxiety comorbidities in people with bipolar disorder. 20% of people with bipolar disorder have a lifetime diagnosis of social anxiety disorder (Pavlova et al. 2015), and 10% of people with bipolar disorder still meet diagnostic criteria for social anxiety disorder when they are euthymic (Pavlova et al. 2017). Several investigations have linked social anxiety disorder with increased rates of suicidal ideation and behaviour (Perroud et al. 2007; Kocabas et al. 2019; Amuk and Patel 2020; Masi et al. 2021), as well as with substance use problems (Yapici Eser et al. 2018) in people with bipolar disorder. These associations were stronger for social anxiety disorder than for any other anxiety disorder (Perroud et al. 2007; Yapici Eser et al. 2018; Kocabas et al. 2019).

While a number of people with bipolar disorder need treatment for social anxiety disorder, the use of some of the treatments is problematic in this population. Antidepressants, specifically the selective serotonin reuptake inhibitors, are the main pharmacological treatment for anxiety (Baldwin et al. 2014; Katzman et al. 2014), including social anxiety disorder (Mayo-Wilson et al. 2014). However, they may destabilize mood in people with bipolar disorder, especially if used without a mood stabilizer (Viktorin et al. 2014; McGirr et al. 2016). Benzodiazepine use is associated with a risk of dependency and negative effects on cognition (Perlis et al. 2010; Cañada et al. 2021). The use of pharmacological treatments for social anxiety disorder is further complicated by the fact that many people with bipolar disorder are already on complex medication regimens. This makes the provision of psychological therapy especially important in this population.

The treatment of choice for social anxiety disorder is individual cognitive behavioural therapy (CBT) (Mayo-Wilson et al. 2014). Please see “Materials and methods” section for a detailed description of the treatment components. The evidence on the efficacy of CBT for social anxiety disorder in people with bipolar disorder is very limited. Bipolar disorder was an exclusion criterion for the studies that informed the recommendation of CBT as the first-line treatment for social anxiety disorder. A previous investigation of group CBT for social anxiety suggested that the magnitude of improvement is not impacted by the comorbid diagnosis of bipolar disorder (Fracalanza et al. 2014). A single case study reported that twenty-three weekly sessions of individual CBT combined with interpersonal psychotherapy led to a remission of social anxiety disorder in one man with bipolar disorder (Queen et al. 2015). However, there are no previous investigations of individual CBT for social anxiety in people with bipolar disorder. In addition, it has been highlighted that data on safety and acceptability of psychological treatments for anxiety in people with bipolar disorder are missing (Stratford et al. 2015). For example, as people with bipolar disorder spend around half of the time with various levels of mood impairment (Judd et al. 2002), mood worsening during a course of CBT is to be expected in some people. The question whether the treatment remains feasible for individuals even when they experience mood deterioration remains unanswered.

We completed a case series to (1) test the acceptability, safety, and efficacy of individual CBT for social anxiety in people who also have bipolar disorder, and to (2) identify any modifications to the standard CBT for social anxiety disorder that may be needed for individuals with bipolar disorder.

## Materials and methods

### Participants

We recruited participants among the outpatients attending the Mood Disorders Program in Halifax, Nova Scotia, Canada. Participants were referred by their treating clinicians. All participants were previously diagnosed with bipolar I or bipolar II disorder and social anxiety disorder by their treating psychiatrists. We included participants with bipolar I or bipolar II disorder who were euthymic and had not changed their medication within the 4 weeks prior to study entry. Euthymia was defined as a score of 12 or less on the Montgomery–Åsberg Depression Rating Scale (Montgomery and Åsberg 1979) and 7 or less on the Young Mania Rating Scale (Young et al. 1978). We required the diagnosis of social anxiety disorder to be one of the main presenting complaints at the time of the assessment to justify the therapeutic focus on it. All diagnoses were confirmed using the Structured Clinical Interview for DSM-5 Disorders (SCID-5) (First et al. 2015). Exclusion criteria were a current diagnosis of a substance use disorder, cognitive impairment that would interfere with the delivery of CBT, being actively suicidal at the time of study entry, and not having a sufficient command of English. We also excluded individuals who were receiving psychological therapy or had a previous adequate course of CBT (at least eight sessions) for social anxiety disorder.

### Procedure

Prior to the study entry, a structured diagnostic assessment was conducted by a research nurse or research assistant using the SCID-5 and agreed in a consensus meeting with the first author (a licensed clinical psychologist). Eligible participants were offered sixteen sessions of CBT for social anxiety within 20 weeks followed by 3 monthly booster sessions. After the sixteenth session, participants met with a research nurse or assistant for the end-of-treatment assessment and then after 3 months for a follow-up assessment. While the standard number of sessions in trials is fourteen, we added two more sessions to allow dealing with depressive symptoms, which we anticipated to occur more frequently in people with bipolar disorder, or to address any social anxiety-related thoughts and images that may specifically occur in people with bipolar disorder.

### Therapy

We used the protocol for individual cognitive therapy for social anxiety disorder (Clark and Wells 1995; Clark et al. 2003, 2006) based on the model by Clark and Wells (1995).

The core components include (1) collaboratively developing a personalized cognitive model of social anxiety; (2) an experiential exercise to demonstrate the negative effects of self-focused attention and safety behaviors, i.e., the self-focused attention and safety behaviours experiment; (3) video and still photo feedback to update negative self-imagery; (4) attention training to practice externally focused attention; (5) behavioral experiments to test patients’ negative beliefs by dropping safety behaviors and focusing attention externally in social situations, and by purposefully displaying feared behaviors or signs of anxiety (decatastrophizing); and (6) developing a therapy blueprint. Additional treatment components include surveys to loosen beliefs alongside experiments; addressing anticipatory worry and post-event rumination; memory work (discrimination training and memory rescripting) to reduce the impact of early social trauma experiences; and additional techniques to address persistent negative self-beliefs and self-criticism. Video illustrations of all treatment techniques are available at www.oxcadatresources.com.

We were open to modifications that may be needed for people with bipolar disorder. All participants were treated by the first author, who is a clinical psychologist and a cognitive behavioural therapist registered with the Canadian Association of Cognitive and Behavioral Therapies (BP). She was supervised by a clinical psychologist and cognitive behavioural therapist registered with the British Association for Behavioural and Cognitive Psychotherapies (EW-P).

### Outcomes

#### Primary outcomes

There were three primary outcomes: (1) Acceptability (defined as the percentage of offered sessions that were attended), (2) safety (defined as the number of severe adverse events related to the treatment, including inpatient admissions, suicide attempts, or other life-threatening events) and (3) the change of the total score on the Social Phobia Inventory (SPIN) (Connor et al. 2000).

While the magnitude of change on the SPIN was the main outcome, for descriptive reasons we also defined remission as a score of 19 or less (Connor et al. 2000). There have been various cut-offs for response on the SPIN. We will report the most stringent cut-off for a response, which is a decrease of at least 50% from baseline (Connor et al. 2000).

#### Secondary outcomes

The secondary outcomes included the total change on The Leibowitz Social Anxiety Scale, self-report version (LSAS) (Baker et al. 2002), the presence or absence of social anxiety diagnosis on the SCID (First et al. 2015), a change in mania severity assessed by the Young Mania Rating Scale (YMRS) (Young et al. 1978), and a change in depression severity measured by the Beck Depression Inventory II (BDI-II) (Beck et al. 1996) and the Montgomery and Åsberg Depression Rating Scale (MADRS) (Montgomery and Åsberg 1979).

Although the overall change on the LSAS was the main secondary outcome, for descriptive purposes we also provide information on the remission and response status on the LSAS. Remission was defined as a score of 30 or less (Rytwinski et al. 2009) and a response as at least a 31% decrease from the baseline (Leichsenring et al. 2013).

## Results

### Participants

Eleven participants took part in the case series. Seven were females and four were males. All participants were outpatients. They ranged in age from nineteen to mid-forties. Eight were White, and three were Asian or Black. Six were in a full-time employment, one was in a part-time employment, three were unemployed, and one was a full-time student. Seven had bipolar I disorder and four had bipolar II disorder. Four had experienced psychotic symptoms during mania. All participants were receiving pharmacological treatment for bipolar disorder. Participants had on average 2.5 current comorbid psychiatric disorders in addition to bipolar disorder. Apart from social anxiety disorder (N = 11), the most common current comorbid psychiatric disorder was generalized anxiety disorder (N = 7), followed by obsessive compulsive disorder (N = 4), body dysmorphic disorder (N = 2), agoraphobia (N = 2), and eating disorders (N = 2).

### Acceptability of CBT for social anxiety disorder in people with bipolar disorder

The participants attended 95% of the offered sessions. Ten participants attended all sixteen sessions and three follow-up appointments. One participant attended nine sessions and no follow-up appointments. This reflects a shorter course of treatment requested by the participant, who attended all research follow-ups. CBT for social anxiety disorder appears to be a highly acceptable treatment for individuals with comorbid bipolar and social anxiety disorder.

### Safety of CBT for social anxiety disorder in people with bipolar disorder

No severe adverse events were reported during the treatment or the 3-month follow-up period. Specifically, there were no suicide attempts and no manic episodes.

### Effect of CBT for social anxiety disorder in people with bipolar disorder

#### Primary outcome

**Fig. 1:**
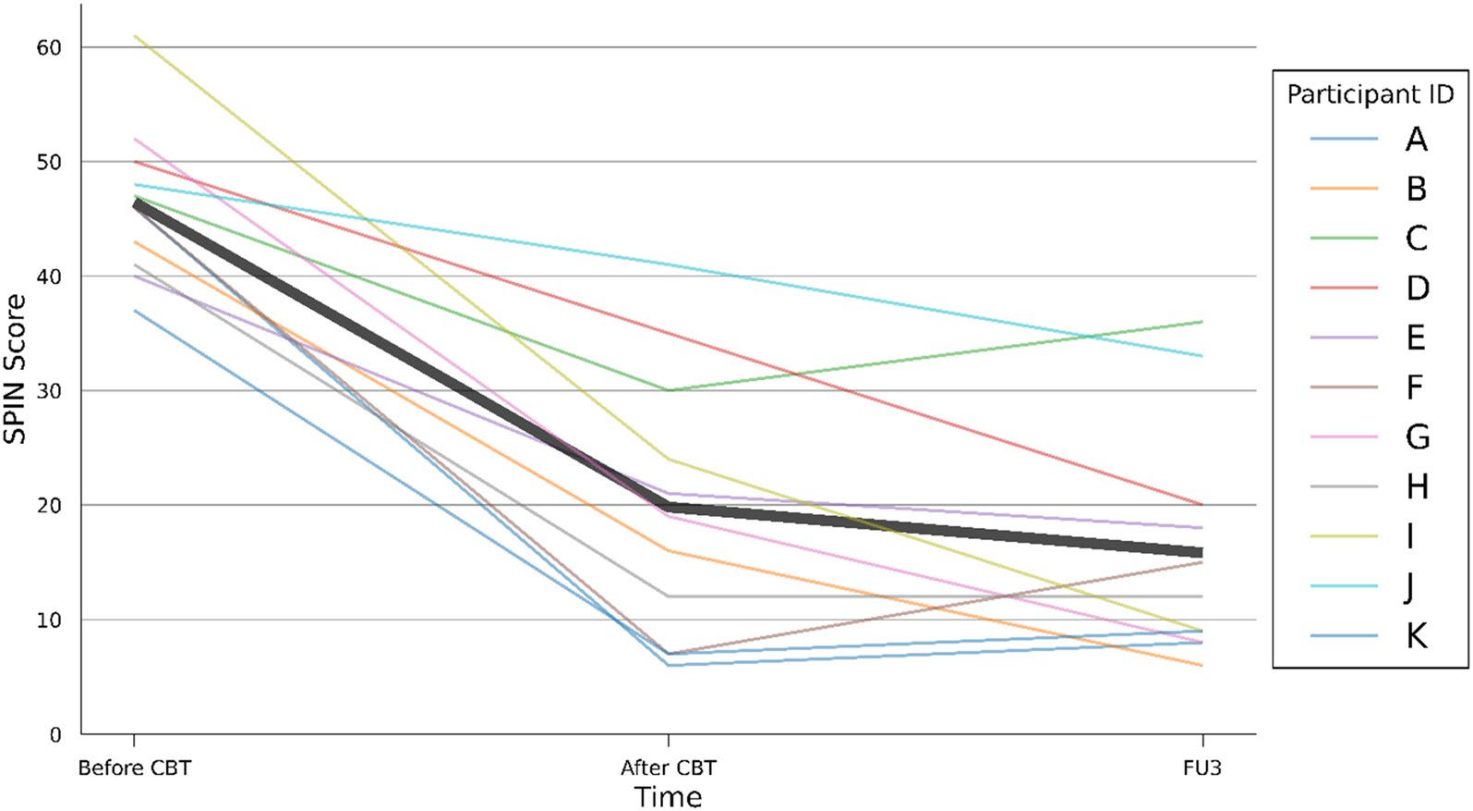
The score on the Social Phobia Inventory (SPIN) before treatment, after treatment and at a 3-month follow-up (FU3). The finer lines denoted as A to K show the SPIN scores of the individual participants. The thick black line is the SPIN average score of all eleven participants

The average score on the SPIN decreased from 46.5 (SD 6.6) before the treatment to 19.8 (SD 11.9) at the end of the sixteen-session intervention and further to 15.8 (SD 10.3) by the end of the 3-month follow-up. Six participants achieved remission by the end of the treatment, one achieved response and four did not meet criteria for treatment response. By the end of the 3-month follow-up, eight individuals achieved remission, one achieved response and two remained non-responders (Fig. 1).

#### Secondary outcomes

##### Social anxiety

**Fig. 2:**
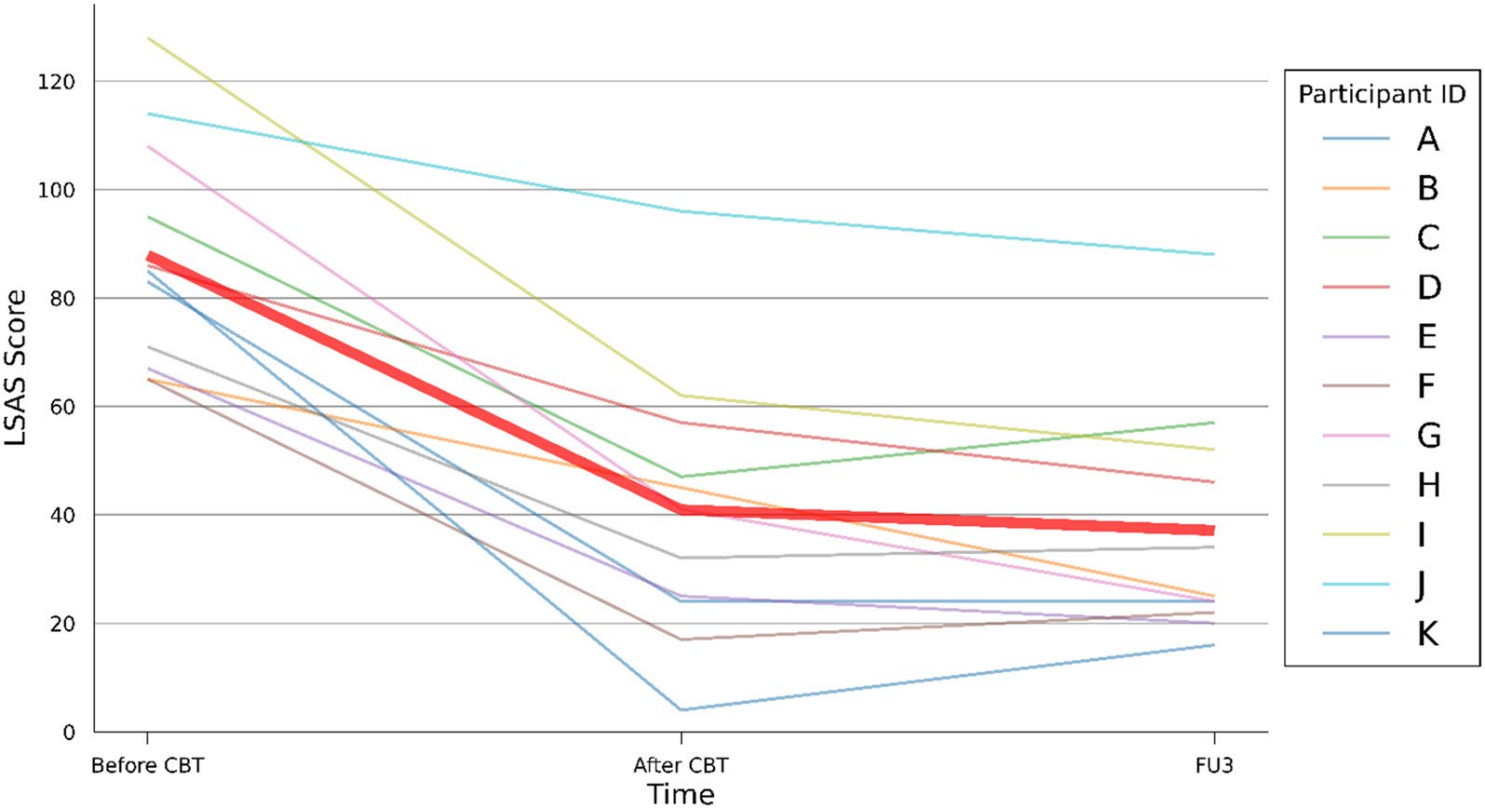
The score on the Leibowitz Social Anxiety Scale Self-Report (LSAS) before treatment, after treatment and at a 3-month follow-up (FU3). The finer lines denoted as A to K show the LSAS scores of the individual participants. The thick red line is the average LSAS score of all eleven participants

The average score on the LSAS halved from 87.9 (SD 21.3) at the beginning to 40.9 (SD 25.2) at the end of the treatment and further decreased to 37.1 (SD 21.8) by the end of the 3-month follow-up. At the end of the treatment, four participants were in remission, five achieved response and two did not respond based on the LSAS score. At the 3-month follow-up, there were six remitted participants, four who responded to treatment and one who did not respond to treatment (Fig. 2).

Ten out of eleven participants no longer met diagnostic criteria for social anxiety disorder at the end of the 3-month follow-up.

##### Mania

The average YMRS score remained low through the course of the study; 2.1 (SD 1.9) at the beginning of the treatment, 1.1 (SD 1.0) at the end of the treatment and 1.2 (SD 2.3) at the end of the 3-month follow-up period. No participant scored above the clinical cut-off during the course of the study.

##### Depression

The average score on MADRS (administered during every encounter) remained in the euthymia range and did not change during treatment; the average score was 5.9 (SD 3.6) at the beginning of the treatment, 5.8 (SD 3.7) at the end of the treatment and 5.1 (SD 4.2) at the end of the 3-month follow-up period. However, with participants’ MADRS scores ranging between 0 and 31 during the treatment, we observed interindividual differences in depression fluctuations. Please see “Relationship between depressive symptoms and improvement in social anxiety” section for more detail.

The average score on BDI-II halved from 12.3 (SD 8.2) at the beginning of the treatment to 6.4 (SD 6.6) at the end of the treatment. At the end of the 3-month follow-up period, the average BDI-II score was 7.6 (SD 8.2).

### Relationship between depressive symptoms and improvement in social anxiety

**Fig. 3:**
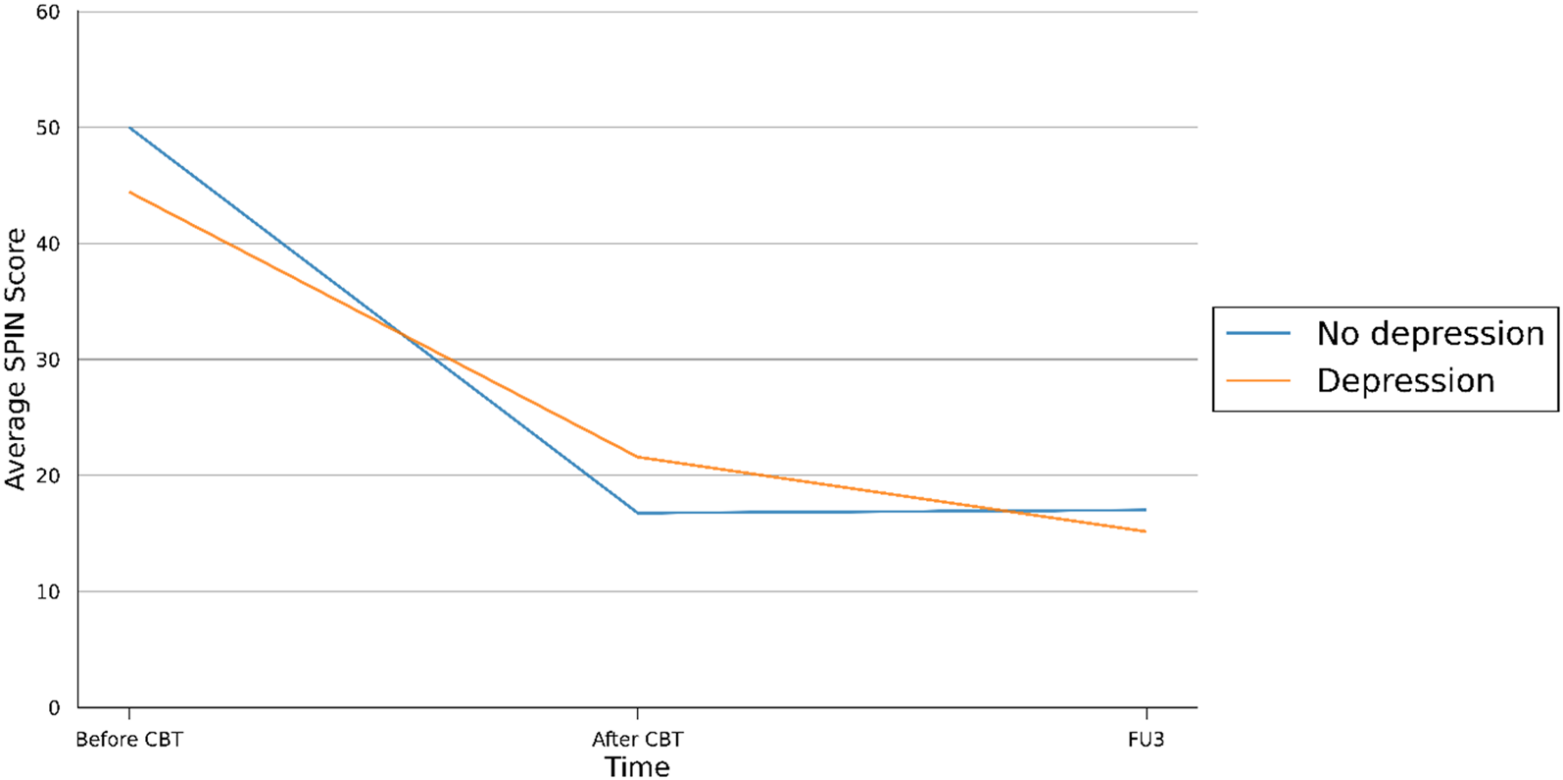
Average score on the Social Phobia Inventory (SPIN) before treatment, after treatment and at a 3-month follow-up (FU3). The blue line is the average SPIN score of the four participants who remained euthymic throughout the treatment. The orange line is the average SPIN score of the seven participants who experienced clinical levels of depression during the treatment

Participants were euthymic at the time of joining the study. However, most participants (seven out of eleven) experienced clinical levels of depression (MADRS˃12) at some point during the treatment. Those who experienced elevated depressive symptoms during the treatment, improved on average by 23 points on the SPIN during the sixteen-session intervention, compared to a 33-point-improvement in the group of people without elevated depressive symptoms. However, at the end of the 3-month follow-up, participants who had experienced elevated depressive symptoms during the treatment improved by further 6 points reaching an overall improvement of 29 points from baseline. The average improvement in the group of participants without significant depressive symptoms during the treatment did not change between the end of treatment and the 3-month follow-up (33 points). Please see Fig. 3.

### Modifications of CBT for social anxiety disorder for people with bipolar disorder

Overall, the treatment did not differ from the standard protocol, including individualized conceptualization, attention and safety behaviours experiment, videofeedback, attention training, behavioural experiments, discrimination training of triggers for social anxiety, imagery rescripting, core belief work, and creating a blueprint. All participants received the core components of CBT for social anxiety disorder.

As is standard for CBT for social anxiety disorder, the focus was on experiential exercises. Participants completed behavioural experiments most sessions during treatment and for homework.

Below, we describe some aspects of CBT for social anxiety disorder that may be specific to people with bipolar disorder.

#### Impact of history of manic and hypomanic episodes on images, thought, and safety behaviors

The negative thoughts and self-images/impressions of some participants in this case series were influenced by their experiences during elevated mood episodes. One participant saw himself as looking “utterly mad” with huge bulging eyes, bright red complexion and saliva flowing from his mouth. Further questioning revealed that this was how he imagined he looked during his manic episode. His safety behaviour was to focus on his face and make sure it moved as little as possible. Another participant was sure that she was being irritating in contact with others. This was based on her experience of people acting irritated when they were unable to interrupt her during her hypomanic episode. Consequently, she avoided social situations as much as possible and when she could not avoid them, she tried to stay away from groups and remain silent. A third participant worried that she may say something offensive like she did during a manic episode. She spent a lot of time preparing “appropriate things to say”. All three participants responded well to interventions, including experiments with dropping safety behaviours to test their fears, feedback from others and videofeedback to update their negative self-images/impression, as well as stimulus discrimination to look for differences between the past manic episode and the present-day social situation.

Another common belief in the participants was “I am weird/different”. This was at least partly based on the participants’ experiences with severe mental illness. As has been emphasized before (Warnock-Parkes et al. 2022), these beliefs were addressed from the very beginning of treatment in behavioural experiments by comparing participants’ predictions with reading the feedback of others, observing their reactions, and watching themselves on the video. Dropping safety behaviours meant to hide this “weirdness” helped participants discover that these were unnecessary and frequently counterproductive. For example, one participant was extremely ashamed about delusional beliefs that she voiced during her manic episode and believed that everyone was able to tell what happened just by looking at her and seeing how “weird” she was. She was trying to blend in by not giving her opinions unless she knew that they were in line with other people’s views, letting others make choices for her, and staying in the background. Consequently, some people stopped asking for her opinions, which strengthened her belief that she was different and had nothing to add. When she started experimenting with voicing her opinions, others were accepting, interested, and started talking to her more. In addition, a decatastrophizing experiment when the participant explicitly disagreed with someone, not only helped her recognize that she was not treated as weird when she disagreed but also that this did not result in a manic episode.

In summary, working with the participants to help them drop their safety behaviours, which were intended to prevent them from appearing or becoming manic, was crucial. By dropping the safety behaviours, the participants were able to find out that that bipolar disorder was not visible to others and that mania could not be triggered by not monitoring themselves in social situations.

#### Dampening

Dampening refers to attempts to decrease the intensity of a positive emotion. Some studies suggest that people with bipolar disorder engage in dampening more often than those without bipolar disorder, possibly because of a fear of mania (Dodd et al. 2019; Edge et al. 2013). They may also avoid rewarding activities in attempts to prevent mania (Edge et al. 2013).

During the treatment, dampening interferes with patients generating generalized positive alternative thoughts (such as “I’m likeable”) from their experiments and with keeping positive data logs. Several participants were worried that noticing positives about themselves may bring on mania. Some participants learned to mistrust any positive thoughts about themselves, because they learned that their grandiose thoughts during mania were inaccurate and led to embarrassment.

Stimulus discrimination was effective in helping participants distinguish between positive self-evaluation occurring during euthymia and overly positive self-impressions that participants experienced during elevated episodes. For example, one participant did not trust any positive thoughts about himself, because he felt he was tricked by his mind before, when he experienced grandiosity during mania. He was able to notice all the differences between the situation during his mania and receiving positive feedback when euthymic (for example, he was now sleeping eight hours per night, he was on effective medication, and he was in a stable job). Socratic questioning about mania triggers was followed by behavioural experiments testing whether writing down positive generalized beliefs about the self on experiment record sheets or keeping a more traditional positive data log would lead to an onset of mania. Work was done on distinguishing between episodes of mania/hypomania and euthymia and understanding the feared behaviours (e.g., saying something offensive) as a symptom of mania or hypomania. Later behavioural experiments with video feedback illustrated the point that the participants did not come across the way they feared. None of the participants experienced mania or hypomania during the treatment.

#### Misdiagnosis danger

A 19-year-old participant was referred for an assessment. One of the referral questions concerned a differential diagnosis between bipolar disorder and schizophrenia due to what appeared like negative symptoms (e.g., no discernable emotional expression, and minimal verbal utterances). Prior to the treatment, this participant experienced a severe manic episode with psychotic features and was hospitalized. Following her discharge, she developed depression and the apparent negative symptoms persevered even after other depressive symptoms improved. A possibility that the apparent negative symptoms were in fact symptoms of social anxiety was raised. During the treatment for social anxiety, it transpired that these were due to the use of safety behaviours (e.g., focusing on how she was coming across and not talking for the fear of being judged). Following the CBT for social anxiety, these symptoms were no longer present. The remission of social anxiety coincided with marked improvement in social and occupational functioning and the participant returned to higher education.

#### Depression

For some participants, a brief detour to depression treatment was used. This mainly focused on activity scheduling and using skills gained in the CBT for social anxiety disorder, including planning behavioural experiments and addressing self-criticism. Some thought records were also used.

## Discussion

Based on this case series, individual CBT for social anxiety delivered during euthymia is an acceptable, safe, and effective treatment for people with bipolar disorder.

Following the treatment, social anxiety in participants with bipolar disorder decreased as much as in samples of people without severe mental illness. The average decrease on the SPIN in our study was identical to that observed in individual CBT delivered in a recent study (Clark et al. 2022). Our secondary social anxiety outcome measure, LSAS, was used in most studies that evaluated individual CBT for social anxiety disorder in people without severe mental illness (Clark et al. 2003, 2006, 2022; Mörtberg et al. 2007; Goldin et al. 2012). The average change on the LSAS in these studies ranged between 29.6 and 52.7. The average change of 47.4 on the LSAS in the current sample of participants with bipolar disorder is at a higher end of this range. Moreover, in keeping with the findings in samples of people without bipolar disorder (Mörtberg et al. 2007; Goldin et al. 2012; Clark et al. 2022), the scores on the social anxiety measures continued to decrease during the follow-up period. At the end of the treatment, five participants were in remission based on the SPIN score and four participants were in remission based on the LSAS score. At the 3-month follow-up, these numbers increased to eight and six as measured by the SPIN and LSAS respectively. This suggests a good maintenance of treatment gains despite the clinical levels of depression that some participants experienced.

Our finding that individual CBT is effective in treating social anxiety in people with bipolar disorder is in line with previous findings on treatments for social anxiety disorder in people with unipolar depression; recent meta-analyses suggested that depressive symptoms or a depressive disorder diagnosis do not adversely affect outcomes of treatments for social anxiety disorder and that in individual CBT people with major depressive disorder may have outcomes superior to those without a mood disorder (Rozen and Aderka 2020).

We only recruited people who were not in a major mood episode at the beginning of the treatment, hence our study was not designed to evaluate the impact of CBT on depressive symptoms, which on average did not change. Despite recruiting only people outside a major mood episode, seven out of eleven participants developed clinical levels of depression during the study. By the end of the sixteen-session treatment, those who experienced depression during the study, improved less than those who remained euthymic. However, by the end of the 3-month follow-up, their treatment gains were comparable to the group who remained euthymic.

The negative association between anxiety and various unfavourable outcomes in people with bipolar disorder is well established (Simon et al. 2007; Lorenzo-Luaces et al. 2018; Pavlova et al. 2018). However, little is known about whether treating anxiety may improve the course of bipolar disorder itself. Among people with bipolar disorder, those with anxiety disorders are more likely to be treated with antidepressants or benzodiazepines (Simon et al. 2004). Treating their social anxiety disorder using individual CBT may simplify their pharmacological regimes and decrease the burden of medication side effects. Future studies should evaluate whether treating social anxiety improves inter-episode functioning and decreases the likelihood of mood episodes relapses.

### Strengths and limitations

This is the first case series that describes the acceptability and efficacy of CBT for social anxiety in people with bipolar disorder during euthymia. We enrolled a similar number of men and women and of people with bipolar I disorder and bipolar II disorder.

It is well established that case series are prone to selection bias (Munn et al. 2020). While we recruited people consecutively, it is possible that participants who were referred by their clinicians and who volunteered to take part were those most likely to respond. Our participants were selected exclusively from a specialist clinic, so may have more severe and complex mood disorders than people with bipolar disorder in general. However, despite greater severity of mood disorders, treatment in specialized mood disorder programs is associated with better long-term outcomes (Kessing et al. 2013). It is not clear whether the findings would generalize to a population treated in a generalist setting.

Additionally, even though the research assessments were not conducted by the therapist, the researchers conducting the assessments were aware of the treatment the participants received. Therefore, we used a self-report measure as the main outcome.

Finally, due to the absence of a comparison group, we cannot exclude a possibility that the participants’ social anxiety would have improved regardless of the treatment. However, the fact that spontaneous remission of social anxiety is unusual in people with bipolar disorder (Pini et al. 2006) makes it unlikely.

The small sample size and the lack of a control group make our findings preliminary.

### Future directions

CBT for social anxiety disorder in people with bipolar disorder needs to be tested in a randomized controlled trial. A trial with a longitudinal follow-up may elucidate not only whether CBT for social anxiety is effective in decreasing anxiety in people with bipolar disorder but could also test whether an improvement in social anxiety serves as a protective factor against future mood episodes.

### Clinical implications

CBT for social anxiety appears to be safe and effective in people with bipolar disorder. It should be available to individuals with bipolar disorder, who also have social anxiety disorder.

Despite enrolling only euthymic participants with bipolar disorder, more than a half developed a transient clinical level of depressive symptoms during the treatment. It was possible to continue the effective treatment with every single participant experiencing depression. Based on this finding, it is likely that people with bipolar disorder presenting with mild to moderate depression are likely to benefit from CBT for social anxiety as long as social anxiety is the primary concern at the time of the enrollment.

Although the standard CBT protocol for social anxiety disorder can be offered to people with bipolar disorder, the treatment providers should be aware that past mood episodes may impact the presentation of social anxiety in people with bipolar disorder. For example, a fear of mania may lead to avoidance of reinforcing stimuli, and the content of social fears may be impacted by experiences from mood episodes.

## Conclusions

CBT for social anxiety disorder in people with bipolar disorder appears safe, acceptable, and effective. Due to the lack of control group and the small sample size in the current investigation, a randomized controlled trial should evaluate CBT for social anxiety disorder in a larger group of people with bipolar disorder.

## Data Availability

The dataset used during the current study is available from the corresponding author on reasonable request.
